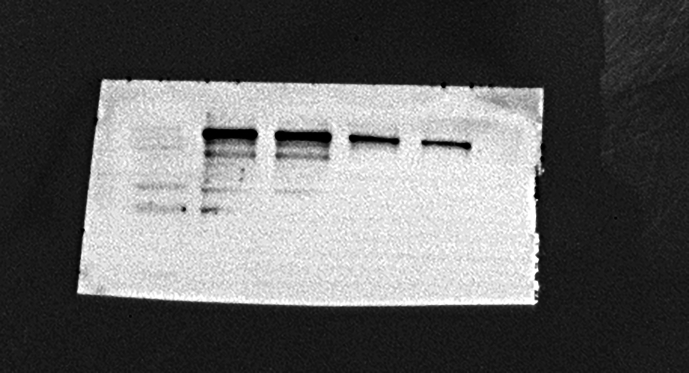# MiR-424-5p suppresses tumor growth and progression by directly targeting CHEK1 and activating cell cycle pathway in Hepatocellular Carcinoma

**DOI:** 10.1016/j.heliyon.2024.e37769

**Published:** 2024-09-11

**Authors:** Chunlin Yin, Yuansong Sun, He Li, Xianxian Zheng

**Affiliations:** a Department of Emergency, The Second Affiliated Hospital of Anhui Medical University,Anhui Hefei, 230601, China; b Research Center of Minimally Invasive Intervention, Anhui Medical University, Anhui Hefei, 230601, China; c Laboratory Department of Hefei First People's Hospital, Anhui Hefei, 230601, China

**Keywords:** Hepatocellular carcinoma, miR-424-5p/CHEK1 axis, Cell cycle pathway

## Abstract

**Objectives:**

The aim of this study is to elucidate the functional mechanism of the miRNA-424-5p/CHEK1 pathway in hepatocellular carcinoma (HCC), thereby offering novel insights for the development of targeted therapeutic strategies for HCC.

**Methods:**

We employed a combination of bioinformatics analysis and data from the GEO to construct a regulatory network between miRNA and mRNA. Real-time quantitative polymerase chain reaction (RT-qPCR) was utilized to assess the expression levels of miR-424-5p and CHEK1. Protein expression of CHEK1 was determined using Western blot analysis. The targeting relationship between miR-424-5p and CHEK1 was validated through a dual-luciferase reporter assay. Furthermore, the effects of miR-424-5p on HCC cell proliferation, migration, and invasion were evaluated using the Cell Counting Kit-8 assay, wound healing assay, and Transwell invasion assay, respectively. Apoptosis of HCC cells was measured by flow cytometry.

**Results:**

Bioinformatics analysis revealed that miR-424-5p was significantly downregulated, while CHEK1 was upregulated respectively in GEO dataset. Furthermore, this inverse expression pattern was observed in both HCC tissues and cell lines. Specifically, downregulation of miR-424-5p was found to promote the proliferation, migration, and invasion of HCC cells, while also inhibiting their apoptosis. The dual-luciferase reporter assay confirmed a direct targeting relationship between miR-424-5p and CHEK1. Inhibition of miR-424-5p was shown to counteract the suppressive effects on HCC cell proliferation, migration, and invasion that result from CHEK1 silencing. Additionally, experimental verification indicated that the activation of the cell cycle pathway is implicated in the oncogenic function of miR-424-5p/CHEK1 in HCC.

**Conclusions:**

The present study demonstrates that miR-424-5p exerts a suppressive effect on HCC cell proliferation, migration, and invasion by downregulating the expression of CHEK1. This finding may offer a theoretical foundation for improving the prognosis and developing novel therapeutic strategies for HCC patients.

## 1 Introduction

Liver cancer is the second most common malignant tumor and ranks as the fourth leading cause of cancer-related deaths worldwide [1,2]. HCC is a type of liver cancer, accounting for 85%–90 % of all primary liver cancers [3]. Patients with early-stage HCC have the option of effective treatments such as surgical resection, liver transplantation, and chemotherapy [4]. However, the treatment for patients with advanced metastatic HCC remains unsatisfactory [5]. Furthermore, the molecular mechanisms underlying HCC recurrence and metastasis are not yet fully understood. As a result, it is a critical issue for clinicians to delve deeper into the pathogenesis of liver cancer and to identify new therapeutic targets that could enhance the efficacy of HCC treatment.

MicroRNAs (miRNAs) are non-coding RNA molecules that are 17–25 nucleotides in length [6] and can negatively regulate gene expression by inducing mRNA degradation or inhibiting translation [7]. A substantial body of research has demonstrated that miRNAs are implicated in the pathogenesis of numerous diseases and are involved in the initiation, progression, metastasis, and drug resistance of various types of cancer [[8], [9], [10], [11]].

Checkpoint kinase 1 (CHEK1) is a serine/threonine protein kinase that plays a pivotal role in the cellular response to DNA damage. When the integrity of the genome is compromised, CHEK1 functions to prevent DNA replication, thereby maintaining genomic stability and preventing abnormal cell division. This action is crucial for inhibiting the formation of tumors. However, in some cancers, including liver cancer, tumor cells have been observed to upregulate CHEK1 [12,13]. This upregulation allows the cells to evade the DNA damage response that would normally be triggered by treatments such as radiotherapy and chemotherapy. As a result, these tumor cells may exhibit increased resistance to drugs and enhanced invasiveness, which poses significant challenges in cancer therapy.

The miR-424-5p has also been implicated in the regulation of various cancers, including its role in liver cancer [14]. While its involvement in cancer development has been noted, the specific mechanism by which miR-424-5p might influence the malignant behavior of liver cancer cells has not been fully elucidated. Specifically, it was previously unknown whether miR-424-5p could target CHEK1 to modulate its expression levels in liver cancer.

Our study sheds new light on this question. We have confirmed that miR-424-5p plays a significant role in the progression of liver cancer. Importantly, our findings indicate that miR-424-5p negatively regulates the expression of CHEK1. By doing so, it inhibits the invasive and proliferative capabilities of liver cancer cells. This discovery not only uncovers a potential regulatory link between miR-424-5p and CHEK1 but also suggests miR-424-5p as a promising molecular target for the development of new therapeutic strategies against liver cancer.

## 2 Materials and methods

### 2.1 Bioinformatics analysis

We obtained HCC-related miRNA transcriptome data (GSE31568, normal: n = 7, tumor: n = 7) and mRNA Counts data (GSE61144, normal: n = 18, tumor: n = 18) from the Gene Expression Omnibus (GEO) [15,16]. Employing the ‘limma’ package in R(4.1.2), we performed differential expression analysis, identifying significantly altered candidates (|logFC|>1.0, adj.P.Val<0.05). With FunRich(3.1.3 http://www.funrich.org/), we predicted target genes of differentially expressed miRNAs (DEMs), crucial for understanding their regulatory impact on HCC. A miRNA-mRNA regulatory network, built from negatively correlated pairs. Subsequently, the mRNAs in the regulatory network were subjected to Gene Ontology (GO) enrichment, Kyoto Encyclopedia of Genes and Genomes (KEGG) pathway analysis, and protein-protein interaction (PPI) network mapping via R(4.1.2) or the STRING database (https://string-db.org/). Key genes were extracted using Cytoscape's (3.9.0) ‘Cytohubba’ plugin and validated through GEPIA(http://gepia.cancer-pku.cn/), confirming their potential as HCC targets.

### 2.2 Specimen collection

HCC tissues and their adjacent non-cancerous liver tissues were collected from 30 patients who had undergone surgical resection at the Second Affiliated Hospital of Anhui Medical University. All HCC cases were pathologically confirmed. Prior to surgery, none of the patients had received any form of systemic anti-cancer therapy, ensuring that the tissues were free from treatment-induced alterations. Immediately following surgical excision, the tissues were snap-frozen in liquid nitrogen to preserve their molecular integrity and then stored at −80 °C to maintain their stability until further analysis.

### 2.3 Cell culture

The human normal liver cell line L-02 and the HCC cell lines HepG2, SMCC7721, Huh-7, and HepG3B were procured from the Institute of Biochemistry and Cell Biology, Shanghai Institutes for Biological Sciences, Chinese Academy of Sciences. These cell lines were cultured in Dulbecco's Modified Eagle Medium (DMEM) (GibcoBRL, Gaithersburg, MD, USA), supplemented with 10 % fetal bovine serum (FBS). The cultures were maintained in a humidified incubator with an atmosphere of 5 % CO2 at 37 °C. Upon reaching 80 %–90 % confluence, the cells were dissociated using 0.25 % trypsin (Shanghai Rugi Biotechnology) for subculturing.

### 2.4 Plasmid construction and cell transfection

The sequences for miR-424-5p and CHEK1 were obtained from the NCBI database. Constructs including the miR-424-5p mimic, miR-424-5p inhibitor, negative control mimic (mimic NC), negative control inhibitor (inhibitor NC), and three distinct siRNAs targeting CHEK1 (si-CHEK1-1#, si-CHEK1-2#, si-CHEK1-3#) as well as a non-targeting siRNA control (si-CHEK1-NC) were synthesized by Shanghai Genepharma Co., Ltd. (Shanghai, China). HepG2 cells were transfected with these constructs using Lipofectamine™ 2000 (Invitrogen, Carlsbad, CA, USA) in accordance with the manufacturer's protocol and then cultured in a humidified incubator with 5 % CO2 at 37 °C. A transfection concentration of 50 nM was applied for all constructs. Cells were maintained in complete growth medium for at least 24 h prior to transfection and washed with phosphate-buffered saline (PBS, pH 7.4) to eliminate serum that might interfere with the transfection process. The efficiency of transfection was evaluated using RT-qPCR, and cells were collected between 24 and 48 h post-transfection for subsequent analysis.

### 2.5 RT-qPCR

Total RNA was extracted from HCC samples and cells using TRIzol reagent (catalog number 15596018, Life Technologies, Carlsbad, CA, USA). The miRNA 1st Strand cDNA Synthesis Kit (tolobio, China) and the ToloScript All-in-one RT EasyMix (tolobio, China) were used to reverse transcribe RNA into cDNA for the amplification of miR-424-5p and CHEK1 genes, following the manufacturers’ instructions. The qPCR reaction mixture, prepared according to the 2 × Q3 SYBR qPCR Master Mix (tolobio, China) protocol, contained 0.4 μL of each primer, 10 μL of 2 × Q3 SYBR qPCR Master Mix, 1 μL of cDNA, and 8.2 μL of ddH2O. The cycling conditions were 95 °C for 1 min, followed by 40 cycles of 95 °C for 20 s and 60 °C for 60 s. The relative expression levels of the target miRNA and mRNA were determined using the 2^−ΔΔCt^ method, where ΔCt = Ct (target gene) - Ct (reference gene-β-actin for CHEK1 and U6 for miR-424-5p), and ΔΔCt = ΔCt (HCC group) - ΔCt (control group). The primer sequences are listed in Table 1.

**Table 1:** Primer sequences for RT-qPCR.

Gene	Forward primer (5′→3′)	Reverse primer (5′→3′)
*β-actin*	CCCTGGAGAAGAGCTACGAG	GGAAGGAAGGCTGGAAGAGT
*U6*	CTCGCTTCGGCAGCACA	AACGCTTCACGAATTTGCGT
*CHEK1*	TGCGTTGTAAGATTTATTTTGGC	TACTTCAGCCCGGTCTTTTT
miR-424-5p	ACACTCCAGCTGGGCAGCAGCAATTCATGT	TGGTGTCGTGGAGTCG

### 2.6 Western blot

Cells were lysed using a radio immunoprecipitation assay lysis buffer (RIPA) from Beyotime (P0013B, Shanghai, China). The lysate was homogenized and centrifuged at 12,000 rpm for 15 min to obtain the supernatant, which contained the total protein. Protein concentration was determined using a bicinchoninic acid (BCA) protein assay kit. Protein samples were then resolved by sodium dodecyl sulfate polyacrylamide gel electrophoresis (SDS-PAGE) and transferred onto a polyvinylidene fluoride (PVDF) membrane. The membrane was blocked with 5 % skimmed milk at room temperature for 2 h to prevent non-specific binding.

Primary antibodies specific to CHEK1 (bs-11226R, Bioss, China), Cyclin B (bsm-52044R, Bioss, China), CDK1 (T55176M, Abmart, China), P53 (bsm-33058M, Bioss, China), phosphorylated P53 (p-P53, ab1431, Abcam, UK), and ATR (bs-23805R, Bioss, China) were applied, followed by incubation with the corresponding secondary antibodies for 1.2 h at room temperature. Detection was performed using an enhanced chemiluminescence (ECL) kit (340958, Thermo Fisher Scientific, Waltham, MA, USA).

The relative expression levels of the target proteins were quantified by analyzing the gray values of the protein bands using ImageJ software (National Institutes of Health, Bethesda, MD, USA). The gray value ratios to GAPDH (TA-08, Zsbio, China) were calculated to normalize the protein expression levels.

### 2.7 Cell Counting Kit 8 (CCK-8) assay

Cells in the logarithmic growth phase were seeded into 96-well plates at a density of 1 × 10^4^ cells per well. The plates were then incubated at 37 °C in a humidified atmosphere containing 5 % CO2 for 12 h. Following treatment for 24 h, 10 μl of CCK-8 reagent (BA00208, Bioss, China) was added to each well. The plates were further incubated for 2 h under the same conditions. Cell viability was assessed by measuring the absorbance at 450 nm using a microplate reader (Infinite M200, Tecan). The absorbance values obtained were used to calculate the percentage of viable cells relative to the control group.

### 2.8 Transwell assays

Cells were adjusted to a concentration of 2 × 10^5^ cells/mL in serum-free medium. An aliquot of 100 μl of this cell suspension was then carefully added to the upper chamber of a transwell insert (Catalog #08416047, Corning, USA). The lower chamber was filled with complete growth medium supplemented with 10 % fetal bovine serum (FBS) to serve as a chemoattractant. The assembled transwell system was incubated for 48 h at 37 °C in a humidified atmosphere containing 5 % CO2.

Following the incubation period, non-migratory cells on the upper surface of the membrane were gently removed using a damp cotton swab. The cells that had migrated to the lower surface of the membrane were fixed with 4 % paraformaldehyde for 30 min at room temperature. Subsequently, the fixed cells were stained with 0.5 % crystal violet solution for 15 min, followed by gentle elution with distilled water to facilitate visualization. The migratory cells on the underside of the upper chamber's membrane were examined using an optical microscope (Olympus, Japan), and representative images were captured.

### 2.9 Wound healing assay

A wound healing assay was conducted to assess the migratory capacity of HepG2 cells. Cells were seeded into 6-well culture plates at a density sufficient to achieve 90 % confluence. Upon reaching the desired confluence, a standardized wound was introduced by scraping the cell monolayer with a 10 μl pipette tip, ensuring uniform wound width across all wells. Following the creation of the wound, the wells were gently washed three times with sterile phosphate-buffered saline (PBS) to remove any detached cells. Fresh complete growth medium was then added to each well, and the cells were allowed to migrate into the wound area for 24 h in a 37 °C, 5 % CO2 incubator.

The migratory response of the cells was monitored by capturing images of the wound area at 0 h and after 24 h of culture using an inverted microscope. The distance migrated by the cells was measured from the images, and the data were used to calculate the rate of cell migration.

### 2.10 Flow cytometry for apoptosis analysis

HepG2 cells were harvested using trypsin without the addition of EDTA. The detached cells were collected and centrifuged at 300×*g* for 5 min at room temperature. Following centrifugation, the supernatant was discarded. Cells were then resuspended in 500 μl of binding buffer. Subsequently, 5 μl of Annexin V-FITC (Catalog #AP101, MULTISCIENCES) was added, followed by the addition of 5 μl of Propidium Iodide (PI). The cells were incubated for 15 min in the dark at room temperature to allow for the binding of the fluorescent probes. Apoptotic cells were quantified using a flow cytometer (CytoFLEX, Beckman Coulter) within 1 h post-staining.

### 2.11 Flow cytometry for cell cycle analysis

After a 48-h transfection period, cells were trypsinized and collected by centrifugation at 300×*g* for 5 min at 4 °C. The supernatant was removed, and the cell pellet was fixed in 70 % ethanol at 4 °C overnight. The fixed cells were then centrifuged again at 300×*g* for 5 min to pellet the cells. The ethanol was aspirated, and the cells were resuspended in 0.5 mL of a staining solution containing PI and RNase A. The cells were incubated at 37 °C for 15 min in the dark to allow for DNA staining. The cell cycle distribution was analyzed by measuring red fluorescence at 488 nm using the same flow cytometer (CytoFLEX, Beckman Coulter). To ensure the reproducibility of the results, the experiment was independently repeated three times.

### 2.12 Dual-luciferase reporter assay

The target plasmids, including miR-424-5p, NC-mimic, CHEK1-3′UTR-WT (wild-type), and CHEK1-3′UTR-Mut (mutant), were transfected into 293T cells. Luciferase activity was assessed 48 h post-transfection using a dual-luciferase reporter assay system. The specific protocol for detection is as follows:

Preparation of Cell Lysate: Dilute the 5 × Passive Lysis Buffer (PLB) with distilled water to a final concentration of 1 × PLB. Add 100 μl of the diluted PLB to each well of a 96-well plate. Cells were lysed by gentle pipetting using a pipette gun, followed by incubation on a room temperature shaking bed for 15 min with slow shaking to ensure thorough lysis.

Collection of Cell Lysate: After lysis, the cell lysate was transferred into a 1.5 ml centrifuge tube and centrifuged at 300×*g* for 10 min at 4 °C to remove cellular debris. The supernatant, containing the soluble proteins, was then transferred to a new tube.

Luciferase Assay: To the 96-well plate, 100 μl of Luciferase Assay Reagent II (LAR II) working solution was added. Subsequently, 20 μl of the prepared cell lysate was added to each well, and the mixture was gently pipetted and mixed 2–3 times to ensure homogeneity. The firefly luciferase activity, serving as the internal control, was measured and recorded.

Renilla Luciferase Assay: Finally, 100 μl of Stop & Glo® Reagent was added to each well. The mixture was again pipetted and mixed 2–3 times, and the Renilla luciferase activity, representing the reporter gene luminescence, was measured and recorded.

This dual-luciferase assay was performed to determine the relative expression levels of the reporter gene under the influence of miR-424-5p and its potential interaction with the CHEK1-3′UTR. To ensure the reproducibility and reliability of the results, the experiment was independently repeated three times.

### 2.13 Tumor xenograft model in BALB/c nude mice

Thirty-six BALB/c nude mice, aged 4 weeks and weighing between 18 and 25 g, were procured from the Experimental Animal Center of the Department of Medicine at Nanchang University (Jiangxi, China). No specific gender was selected for this study. HepG2 cells (3.0 × 10^6^) were stably transfected with either lentivirus-miR-424-5p (LV-miR-424-5p) or lenti-MOCK. These cells were then subcutaneously injected into the lower flank region of the mice. The anesthesia was administered using pentobarbital sodium at a dosage of 0.1–0.2 ml per 10 g of body weight, following the guidelines provided by the China Laboratory Animal Information Network.

The lentivirus used for transfection was sourced from Shanghai GenePharma (Shanghai, China). Ten days post-inoculation, the tumor volume was measured and calculated using the formula: Volume = (Length × Width^2^)/2 (units in mm^3^). At the end of the 6-week experimental period, the mice were humanely euthanized using carbon dioxide asphyxiation, and the tumors were surgically excised and weighed. The data obtained were used to plot a curve representing the average tumor volumes at various time points throughout the study. This xenograft model was utilized to assess the effect of miR-424-5p on tumor growth *in vivo*.

### 2.14 Statistical analysis

All statistical analyses were conducted using SPSS software version 26.0 (IBM Corporation, Armonk, NY, USA). Graphical representations of the data were generated using GraphPad Prism software version 9.0 (GraphPad Software, La Jolla, CA, USA). Data are presented as the mean ± standard deviation (SD). To determine the statistical significance of differences between two groups, a Students t-test was employed. A p-value of less than 0.05 was considered to indicate statistical significance.

## 3 Results

### 3.1 Construction of the miRNA-mRNA regulatory network and screening of hub genes

The datasets GSE31568 and GSE61144 were analyzed using R software (version 4.1.2), resulting in the identification of 209 differentially expressed miRNAs (DEMs) and 173 differentially expressed genes (DEGs) (Fig. 1A–B). The miRNA-mRNA regulatory network was constructed based on the principle of negative regulation between miRNAs and their target mRNAs (Fig. 1C). Subsequently, the protein-protein interaction (PPI) network analysis of DEGs was conducted utilizing the STRING database, revealing that CHEK1 occupied a central hub position within the PPI network (Fig. 1D). Further investigation using the GEPIA database indicated that the overexpression of CHEK1 was associated with poor prognosis in HCC (Fig. 1G–H). Consequently, we resolved to further validate the miR-424-5p/CHEK1 regulatory axis both *in vivo* and *in vitro*, and to explore its impact on the cell cycle signaling pathway.

**Fig. 1:**
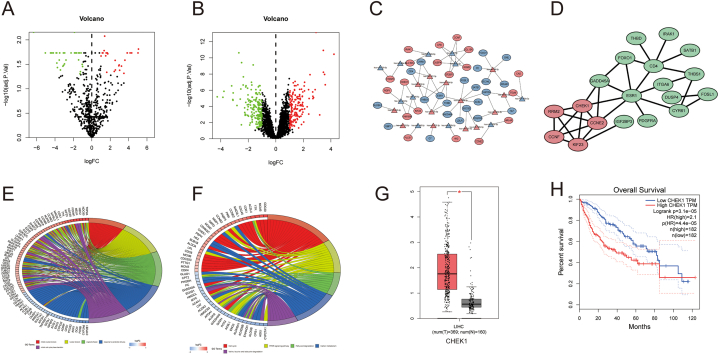
**The results of Bioinformatics analysis. A-B:** Volcano plot of DEMs and DEGs in normal group and tumor group of HCC from GEO database; **C:** miRNA-mRNA regulatory network; **D:** PPI network analysis of DEGs**; E-F:** The result of GO and KEGG analysis;**G:** The expression level of CHEK1 in liver cancer and non-tumor tissues from TCGA database; **H:** Kaplan-Meier analysis of overall survival from TCGA-LIHC cohort based on CHEK1 expression.

### 3.2 CHEK1 is highly expressed, while miR-424-5p is poorly expressed in HCC tissues and cell lines

The expression levels of miR-424-5p and CHEK1 in HCC tissues and adjacent normal tissues, collected from 30 HCC patients, were determined using RT-qPCR. The results showed that miR-424-5p was less expressed, whereas CHEK1 was more highly expressed in HCC tissues compared to adjacent normal tissues (Fig. 2A–B). Additionally, we detected the expression levels of miR-424-5p and CHEK1 in the normal cell line L-02 and various HCC cell lines using RT-qPCR. As shown in Fig. 2C–D, the expression levels of miR-424-5p in the HCC cell lines were significantly lower, with the following order: HepG2, HepG3B, HuH-7, SMMC-7721, and L-02. Conversely, CHEK1 expression levels were significantly higher in the HCC cell lines, with the following order: HepG2, HepG3B, HuH-7, SMMC-7721, and L-02. Therefore, the HepG2 HCC cell line was chosen for subsequent experiments.

**Fig. 2:**
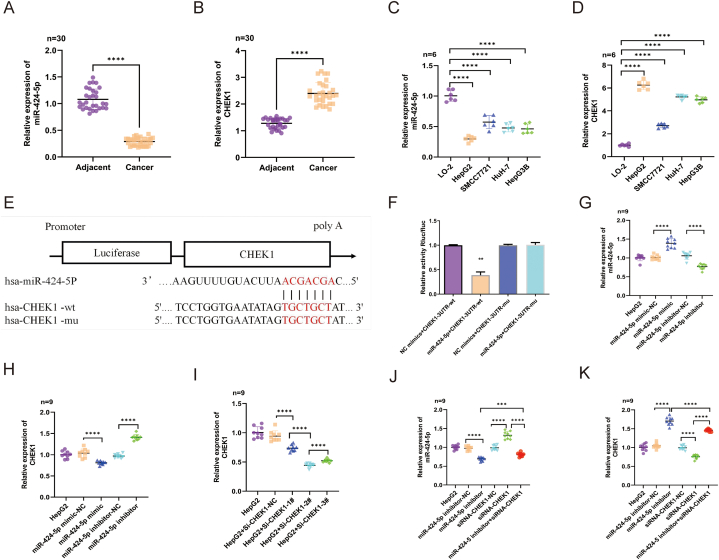
**MiR-424-5p negatively regulates the expression of CHEK1**:**A-B:** The expression levels of MiR-424-5p and CHEK1 in HCC tissues and adjacent tissues;**C-D:** The expression of MiR-424-5p and CHEK1 in HCC cell lines analyzed by RT-qPCR; **E:** The binding sites between MiR-424-5p and CHEK1 were predicted by Targetscan database; **F:** Dual-luciferase reporter assay was used for the evaluation of the luciferase activities of the luciferase reporter vectors CHEK1 3UTR-WT and MUT in HepG2 cells transfected with miR-424-5p or miR-NC, respectively; **G-H:** Effects of miR-424-5p upregulation or downregulation on miR-424-5p and CHEK1 mRNA expression of HeG2 cells were examined via RT-qPCR. miR-424-5p-minic-NC and miR-424-5p-inhibitor-NC were the control group; **I:** RT-qPCR was executed to assess CHEK1 mRNA expression in HeG2 cells transfected with si-CHEK1#1, si-CHEK1#2,si-CHEK1#3 or si-NC; HepG2 + si-CHEK1-NC was the control group; **J-K:** Expression of miR-424-5p or CHEK1 in miR-424-5p and CHEK1 suppressed HeG2 cells was examined by RT-qPCR; miR-424-5p-inhibitor-NC and siRNA CHEK1-NC were the control group; **P < 0.01 and ****P < 0.0001.

### 3.3 MiR-424-5p can act as a gene expression regulator by binding to the 3′UTR of CHEK1

We identified a binding site between miR-424-5p and CHEK1 using FunRich software. Subsequently, a dual-luciferase reporter assay was conducted to assess the regulatory interaction between miR-424-5p and CHEK1. As depicted in Fig. 2E–F, the luciferase activity of the CHEK1 wild-type (WT) construct was significantly suppressed in the presence of the miR-424-5p mimic (p < 0.05), whereas the activity of the CHEK1 mutant (MT) construct did not change significantly (p > 0.05). Further studies involved RT-qPCR and Western blot analyses in HCC cell lines treated with miR-424-5p mimic or inhibitor, as well as in control cells. The results showed that CHEK1 expression was significantly reduced in the miR-424-5p mimic-treated cells, while it was markedly elevated in the miR-424-5p inhibitor-treated cells (Fig. 2H). Additionally, we observed that the miR-424-5p expression level was significantly higher in the si-CHEK1 group compared to the si-CHEK1-NC group, and its expression was significantly reduced upon co-transfection with miR-424-5p inhibitor and si-CHEK1 (Fig. 2J–K). These findings suggest that CHEK1 is a target gene of miR-424-5p.

### 3.4 The effects of miR-424-5p and CHEK1 on the proliferation, migration, and invasion of HepG2 cells were investigated

Initially, RT-qPCR analysis was conducted to assess the transfection efficiency and expression levels of miR-424-5p mimic/inhibitor(Fig. 2G) and three specific siRNAs targeting CHEK1 (si-CHEK1-1#, si-CHEK1-2#, and si-CHEK1-3#) in HepG2 cells. Among the siRNAs, si-CHEK1-2# demonstrated the lowest expression level of CHEK1, followed by si-CHEK1-3#, si-CHEK1-1#, and the negative control (NC) or untransfected cells (Fig. 2I). Therefore, si-CHEK1-2# was chosen for subsequent experiments.

The CCK-8 assay, wound healing assay, and transwell assay were employed to evaluate the impact of miR-424-5p and CHEK1 on HepG2 cell proliferation, migration, and invasion, respectively. Transfection with the miR-424-5p inhibitor significantly enhanced the proliferation, migration, and invasion of HepG2 cells compared to the matched controls (Fig. 3A–B,E-G). No significant difference was observed between the control group and the inhibitor-NC group. Conversely, transfection with si-CHEK1 resulted in suppressed proliferation, migration, and invasion compared to the matched controls(Fig. 3A–B,E-G). No significant difference was noted between the control group and the si-NC group. Furthermore, the inhibitory effects of CHEK1 silencing on HepG2 cell proliferation, migration, and invasion were reversed by the miR-424-5p inhibitor(Fig. 3A–B,E-G).

**Fig. 3:**
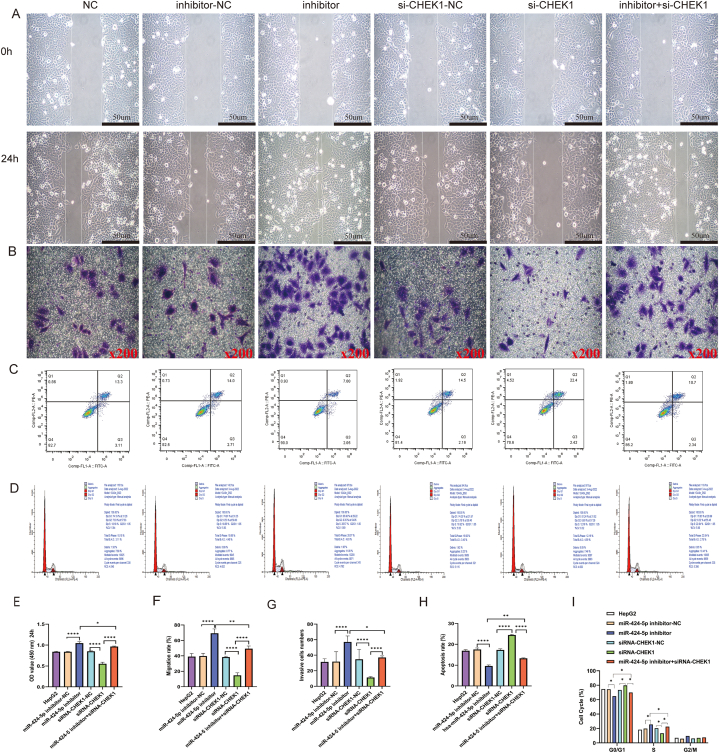
MiR-424-5p regulated CHEK1 to affect proliferation, migration, invasion, and apoptosis of HCC cells. HepG2 cells were co-transfected with miR-424-5p inhibitor or inhibitor NC and si-CHEK1 or si-NC and miR-424-5p inhibitor + si-CHEK1:**A and F:** Cell migration abilities were checked by wound healing assay; miR-424-5p-inhibitor-NC and siRNA CHEK1-NC were the control group; **B and G:** Cell invasion abilities were checked by transwell assay; miR-424-5p-inhibitor-NC and siRNA CHEK1-NC were the control group; **C and H:** Cell apoptosis was determined by flow cytometry assay; miR-424-5p-inhibitor-NC and siRNA CHEK1-NC were the control group; **D and I:** Cell Cycle was determined by flow cytometry assay; miR-424-5p-inhibitor-NC and siRNA CHEK1-NC were the control group; **E:** Cell proliferation abilities were determined by CCK-8 assay; miR-424-5p-inhibitor-NC and siRNA CHEK1-NC were the control group; *P < 0.05, **P < 0.01 and ****P < 0.0001.

### 3.5 The effects of miR-424-5p and CHEK1 on the apoptosis and cell cycle in HepG2 cells

To elucidate the effects of miR-424-5p and CHEK1 on HepG2 cells, apoptosis and cell cycle analysis were conducted on cells transfected with various plasmids, including inhibitor-NC, miR-424-5p inhibitor, si-CHEK1-NC, si-CHEK1, and a combination of miR-424-5p inhibitor and si-CHEK1. Flow cytometry was utilized to assess these cellular processes.

The results indicated that, compared to the inhibitor-NC group, the miR-424-5p inhibitor group had a reduced proportion of cells in the G1 phase, an increased proportion in the S phase, and a decreased rate of apoptosis (Fig. 3C–D, H-I). In contrast, silencing CHEK1 led to an accumulation of cells in the G1 phase, a decrease in the S phase population, and an elevated rate of apoptosis (Fig. 3C–D, H-I). There was no significant difference between empty plasmid group and si-NC group (Fig. 3C–D, H-I). Additionally, the inhibition of miR-424-5p was found to alleviate cell cycle arrest and rescue the apoptosis induced by si-CHEK1(Fig. 3C–D, H-I). Collectively, these findings suggest that miR-424-5p silencing restrains apoptosis in HepG2 cells, whereas CHEK1 silencing promotes it.

### 3.6 Activation of the cell cycle pathway is implicated in the oncogenic functions of the miR-424-5p/CHEK1 regulatory axis in HCC

KEGG enrichment analysis revealed that CHEK1 is significantly enriched in the cell cycle pathway in HCC (Fig. 1E–F). To further elucidate the molecular mechanisms underlying its functional role in HCC, Western blot analysis was conducted to examine proteins associated with the cell cycle pathway. As depicted in Fig. 4A–B, compared to the NC group, the expression levels of Cyclin B, CDK1, p-P53, and ATR were found to be differentially regulated in the miR-424-5p inhibitor and si-CHEK1 groups *in vitro*. These findings suggest that the miR-424-5p/CHEK1 regulatory axis contributes to HCC tumorigenesis, at least in part, by activating the cell cycle signaling pathway.

**Fig. 4:**
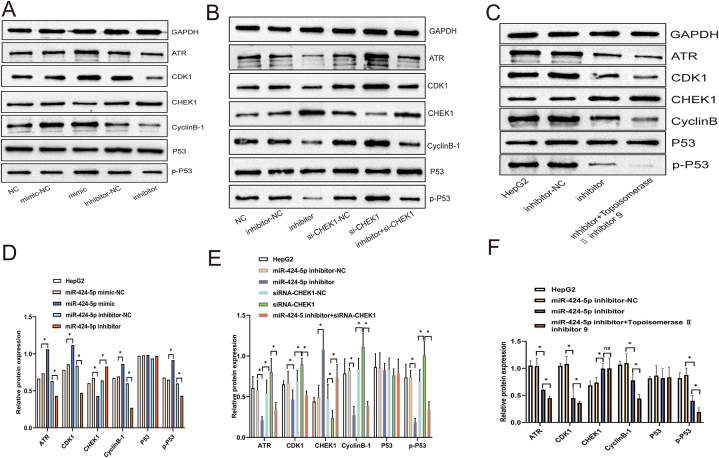
**The Mir-424-5p/CHEK1 regulatory axis may affect the cell cycle signaling pathway *in vivo* or *in vitro*.** The expression of GAPDH,ATR,CDK1,CyclinB, P53,p-P53 was determined by Western blot. **A and D:** HepG2 cells were co-transfected with miR-424-5p mimic or mimic NC and miR-424-5p inhibitor or inhibitor NC *in vitro*; miR-424-5p-minic-NC and miR-424-5p-inhibitor-NC were the control group; **B and E:** HepG2 cells were co-transfected with miR-424-5p inhibitor or inhibitor NC,si-CHEK1 or si-CHEK1 NC and miR-424-5p inhibitor + si-CHEK1 *in vitro*; miR-424-5p-inhibitor-NC and siRNA CHEK1-NC were the control group; **C and F:** HepG2 cells were co-transfected with miR-424-5p inhibitor or inhibitor NC and miR-424-5p inhibitor + Topoisomerase Ⅱ inhibitor 9 *in vivo*; miR-424-5p-inhibitor-NC was the control group; *P < 0.05.

### 3.7 miR-424-5p negatively regulates CHEK1 and impacts the development of HCC via the cell cycle pathway

To further explore the effect of miR-424-5p on HCC proliferation, a nude mice xenograft tumor model was utilized. Nude BALB/c mice were xenografted with a HepG2 stable cell line overexpressing miR-424-5p. A significant reduction in miR-424-5p expression was observed in the inhibitor group (Fig. 5C), which was associated with an increase in tumor size and weight compared to the control HepG2 cells or NC group (Fig. 5A–B, E). Western blot analysis showed a significant decrease in the expression of proteins related to the cell cycle signaling pathway in the inhibitor group compared to the control group (Fig. 4C–F). Importantly, the application of the cell cycle pathway inhibitor topoisomerase II further enhanced this difference(Fig. 4C–F). Collectively, these results suggest that miR-424-5p can suppress HCC proliferation *in vivo*, at least in part, by regulating the cell cycle pathway.

**Fig. 5:**
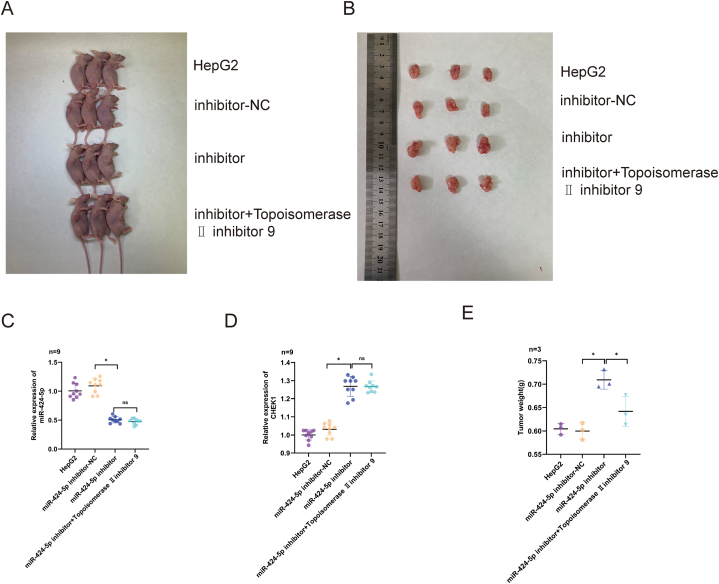
**Impacts of miR-424-5p suppression on HepG2 cell growth *in vivo*.** HepG2 cells were co-transfected with miR-424-5p inhibitor or inhibitor NC and miR-424-5p inhibitor + Topoisomerase Ⅱ inhibitor 9:**C-D:** The expression levels of MiR-424-5p and CHEK1; miR-424-5p-inhibitor-NC was the control group; **A-B and E:** Tumor size or weight in the mice of the different experimental groups was assessed on day 35 after injection; miR-424-5p-inhibitor-NC was the control group; *P < 0.05.

## 4 Discussion

At present, liver cancer is the fourth leading cause of cancer related deaths in the world, and the number of deaths is increasing year by year [17]. About 90 % of liver cancer patients are HCC [18]. Although the comprehensive treatment strategy of liver cancer has benefited some patients, however, the prognosis of liver cancer patients is still poor [19]. Therefore, it is particularly important to find new therapeutic targets for liver cancer for patients with poor surgical treatment effect or anti-tumor drug resistance. This study explored the new molecular mechanism of liver cancer progression, to provide a theoretical basis for the development of new therapeutic targets for liver cancer.

MicroRNAs (miRNAs), as pivotal noncoding RNAs, have been demonstrated to influence the biological behaviors of numerous tumors, including proliferation, apoptosis, migration, and invasion [20,21]. Among them, miR-424-5p has emerged as a tumor suppressor with significant implications in various human cancers. Its dysregulation in malignant tumors is strongly correlated with the initiation and progression of cancer. In thyroid cancer, overexpression of miR-424-5p is significantly linked to distant metastasis [22]. In breast cancer, diminished levels of miR-424-5p are associated with advanced clinical staging, increased tumor volume, a higher metastatic lymph node count, the presence of distant metastases, and a lower histological grade [23]. Recent findings suggest that miR-424-5p may promote endometrial cancer progression by modulating the CLDN4/PI3K/AKT signaling pathway [24]. Additionally, in colorectal cancer, miR-424-5p has been shown to enhance cell proliferation and metastasis by directly targeting SCN4B [25]. Previous studies have established that miR-424-5p can suppress tumor biological behaviors in liver cancer, while CHEK1 promotes the proliferation, migration, and invasion of liver cancer cells. However, the regulatory relationship between miR-424-5p and CHEK1 in HCC remains elusive. Utilizing bioinformatics analysis of the GEO database, we identified differential expression of miR-424-5p and CHEK1 in liver cancer tissues. Our subsequent experiments using RT-qPCR confirmed these findings in HCC tissues and multiple HCC cell lines. Functional assays, along with FunRich software predictions and dual-luciferase reporter experiments, demonstrated that miR-424-5p binds to the 3′UTR of CHEK1, negatively regulating its expression at the post-transcriptional level. This regulation was further validated by RT-qPCR and Western blot analysis. Through cell functional experiments, we explored the specific molecular mechanism of the miR-424-5p/CHEK1 regulatory axis, providing insights into the pathogenesis of HCC.

The results of the cell function experiment demonstrate that the proliferation, migration, and invasion of HepG2 cells were significantly enhanced, the apoptosis was reduced, the number of cells staying in G1 phase was reduced, and more cells stayed in S phase after transfection with miR-424-5p inhibitor compared with NC group and inhibitor NC group. On the contrary, silencing CHEK1 can significantly inhibit the proliferation, migration, and invasion of HepG2 cells, and significantly increase apoptosis. The rescue experiment further showed that inhibition of CHEK1 could partially reverse the effect of miR-424-5p-inhibitor on the biological behavior of HepG2 cells.

In addition, we also conducted nude mouse tumorigenesis experiments and found that miR-424-5p inhibitor can significantly promote tumor growth, and the expression level of CHEK1 is significantly increased, which indicate that miR-424-5p, as a tumor suppressor gene for HCC, plays a regulatory role by negatively regulating the expression of CHEK1. To further explore the molecular mechanism of the influence of miR-424-5p/CHEK1 regulatory axis on HCC, we also conducted functional enrichment analysis on DEGs and found that CHEK1 was mainly enriched in the cell cycle signaling pathway. Subsequently, we detect the alteration of cell cycle signaling pathway related proteins through Western blot analysis. As shown in the figure, with changes in the expression levels of miR-424-5P and CHEK1, the levels of cell cycle signaling pathway related proteins (such as Cyclin B, CDK1, p-P53, and ATR) also changed. Based on the results of cell cycle analysis, we speculate that the miR-424-5p/CHEK1 regulatory axis can further affect the proliferation, migration, invasion, and apoptosis of HCC cells through the cell cycle signaling pathway.

In summary, our study underscores the clinical and biological relevance of the miR-424-5p/CHEK1 regulatory axis in HCC, revealing its critical downstream influence on the cell cycle pathway. This axis exerts a pivotal control over tumorigenesis and progression through the modulation of cellular proliferation and cell cycle regulation, with significant repercussions on the cell cycle pathway. While prior studies have noted the irregular expression patterns of miR-424-5p and CHEK1 in HCC, our research presents the first comprehensive elucidation of miR-424-5p′s direct inhibitory effect on CHEK1 in HCC. Furthermore, our bioinformatics analysis unveils a potentially broader regulatory network, where miR-424-5p may also govern the expression of additional target genes, including RGP1, FAM189B, and KIF23. Intriguingly, CHEK1 expression in liver cancer cells appears to be under the dual regulation of miR-424-5p and miR-199-5p. These insights not only spotlight the intricate pathophysiology of HCC but also chart new avenues for future investigative pursuits.

## Ethics approval and consent to participate

This study was approved by the ethics commitee of the Second Affiliated Hospital of Anhui Medical University(YX-2022-038F2,Approval date:2022.6.23). Written informed consent was obtained from all the participants prior to the enrollment of this study.

## Consent for publication

Written informed consent was obtained from the patient for publication of this case report and any accompanying images. A copy of the written consent is available for review by the Editor-in-Chief of this journal.

## Funding

This work was supported by the Clinical Fund of 10.13039/501100002947Anhui Medical University (Grant No. 2021xkj178)， the Basic and Clinical Collaborative Research Enhancement Program Project of 10.13039/501100002947Anhui Medical University (Grant No. 2022xkjT034).

## Data availability statement

The datasets generated or analyzed during this study areavailable from the corresponding author on reasonable request.

## CRediT authorship contribution statement

**Chunlin Yin:** Writing – original draft, Resources, Methodology, Conceptualization. **Yuansong Sun:** Data curation. **He Li:** Writing – review & editing, Supervision, Methodology, Conceptualization. **Xianxian Zheng:** Writing – review & editing, Supervision, Methodology, Conceptualization.

## Declaration of competing interest

The authors declare that they have no known competing financial interests or personal relationships that could have appeared to influence the work reported in this paper.

## Figures and Tables

**figs1:**
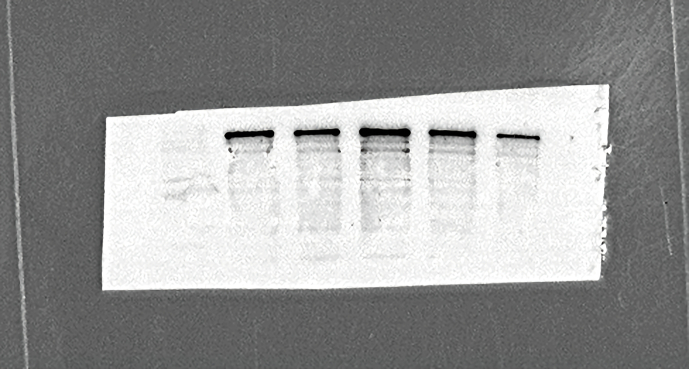


**figs2:**
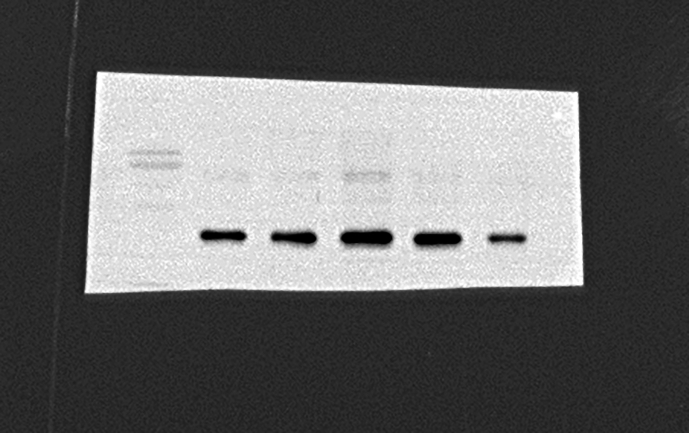


**figs3:**
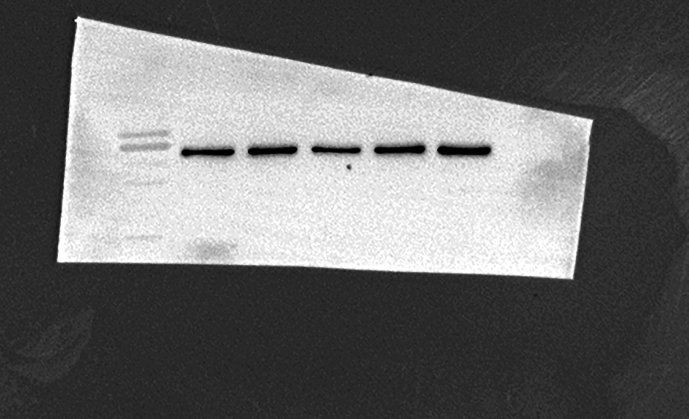


**figs4:**
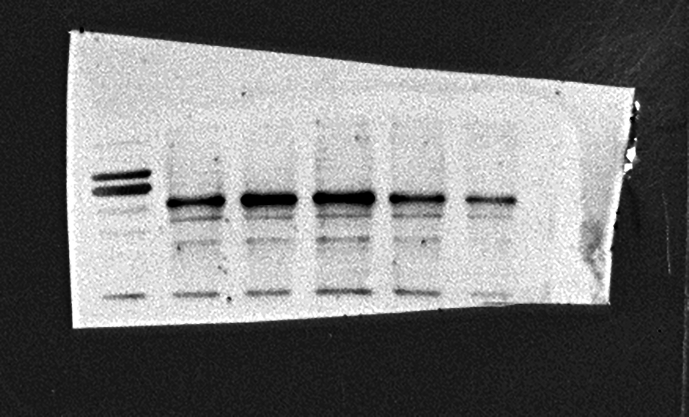


**figs5:**
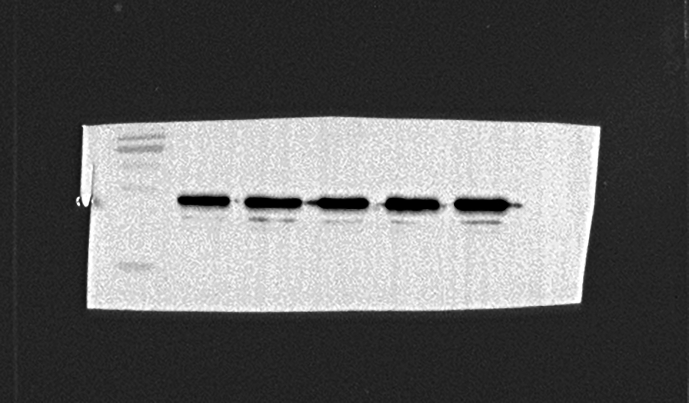


**figs6:**
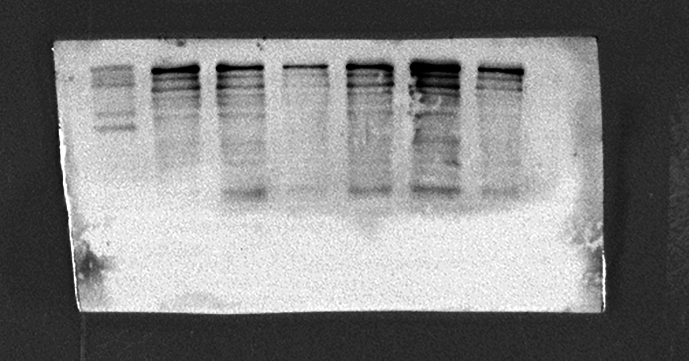


**figs7:**
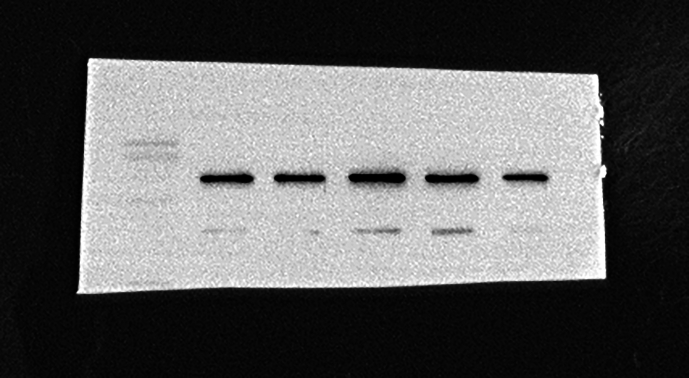


**figs8:**
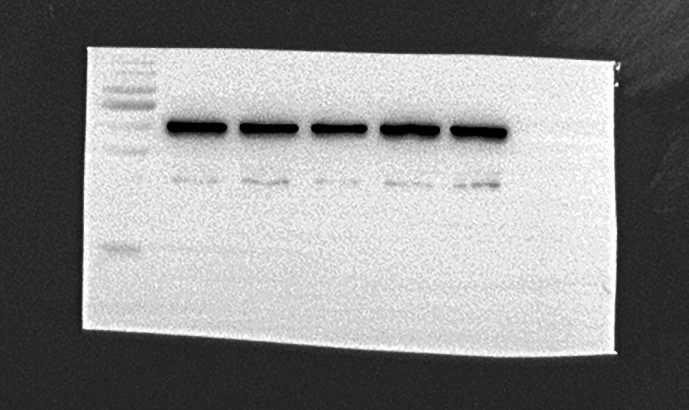


**figs9:**
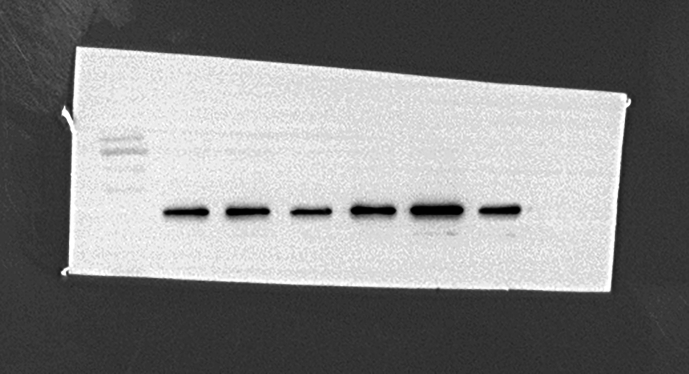


**figs10:**
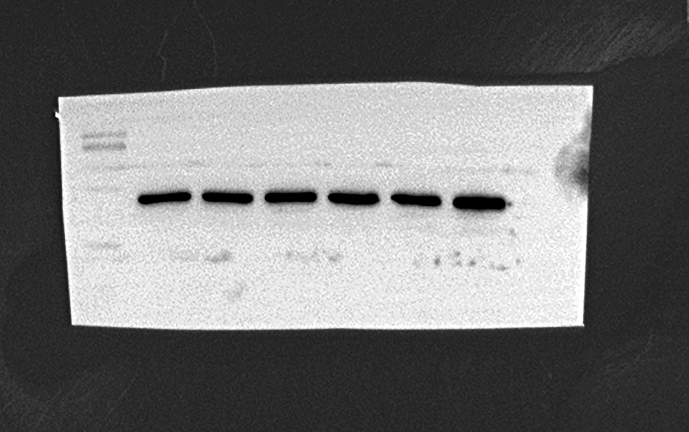


**figs11:**
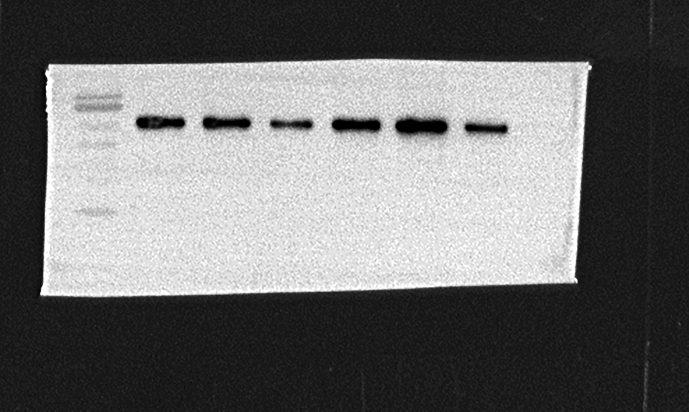


**figs12:**
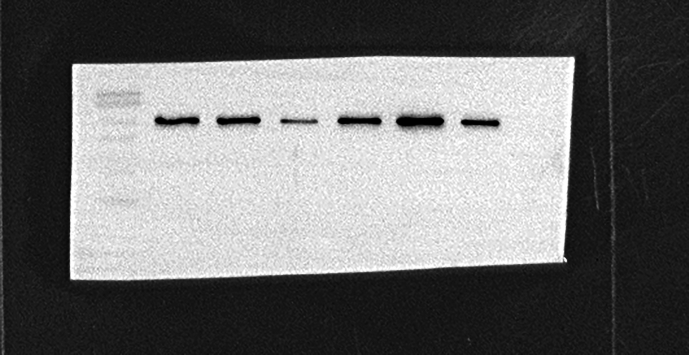


**figs13:**
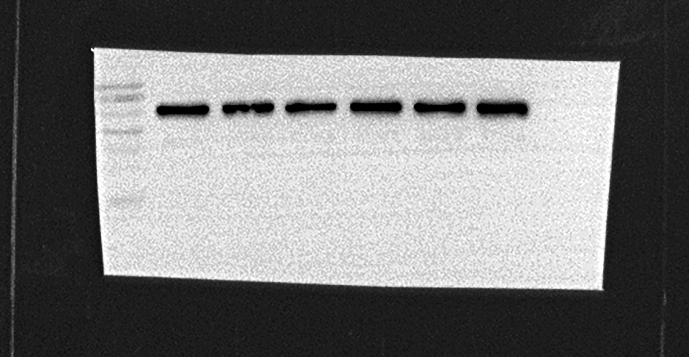


**figs14:**
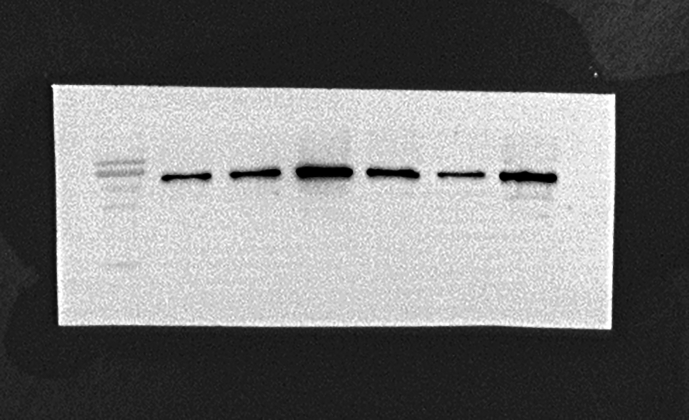


**figs15:**
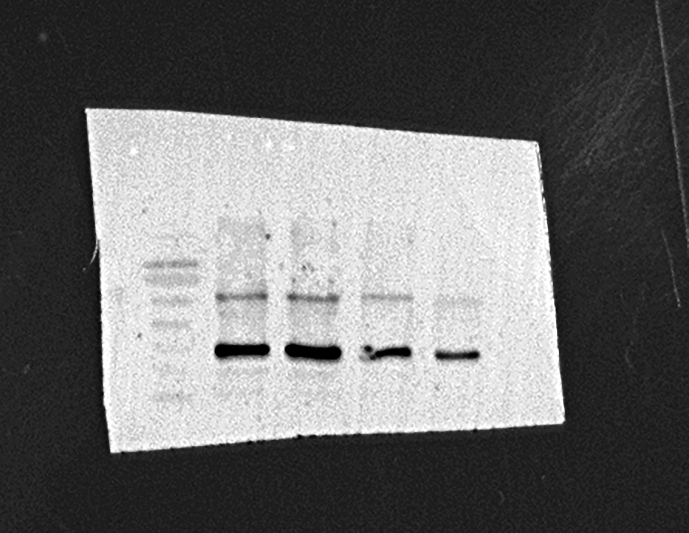


**figs16:**
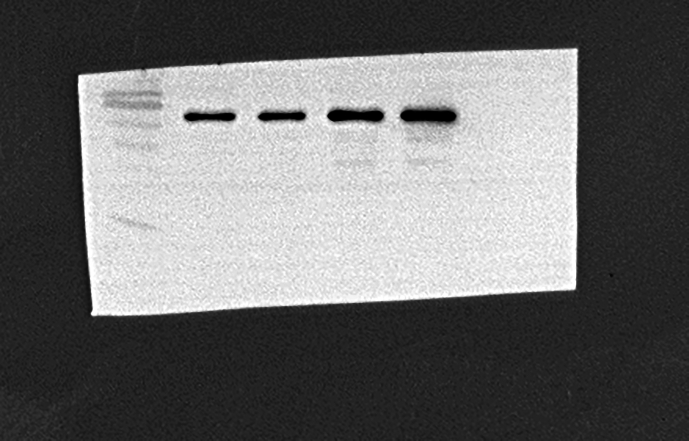


**figs17:**
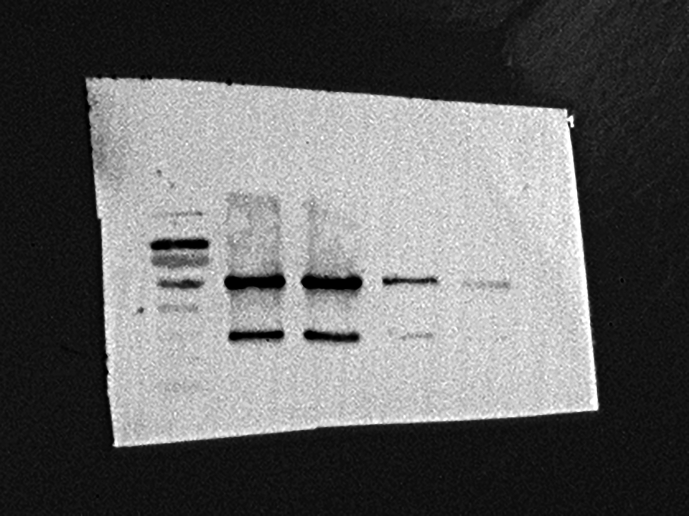


**figs18:**
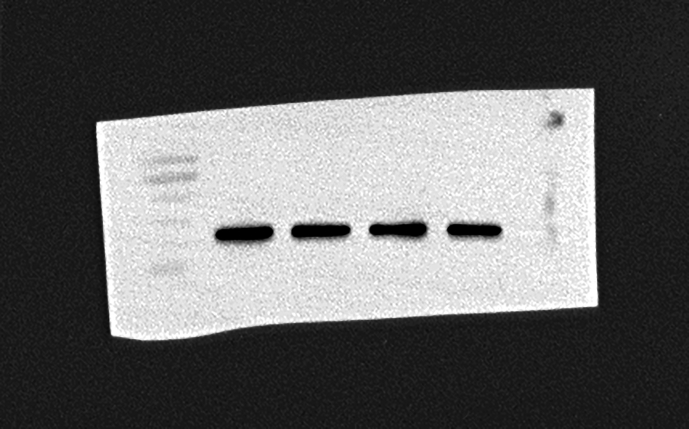


**figs19:**
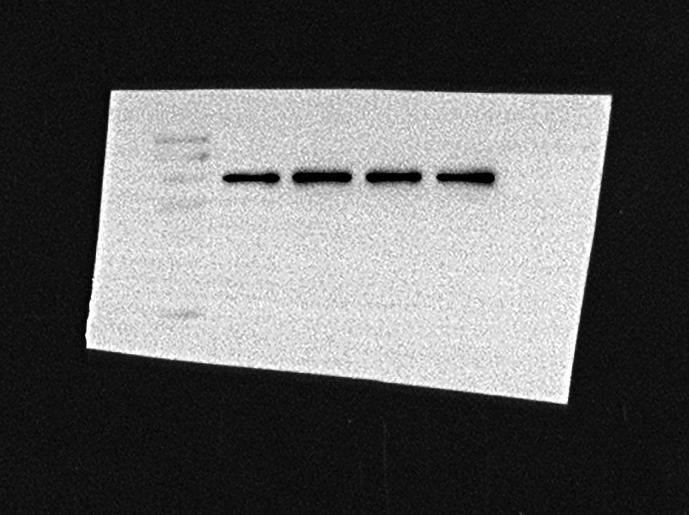


**figs20:**
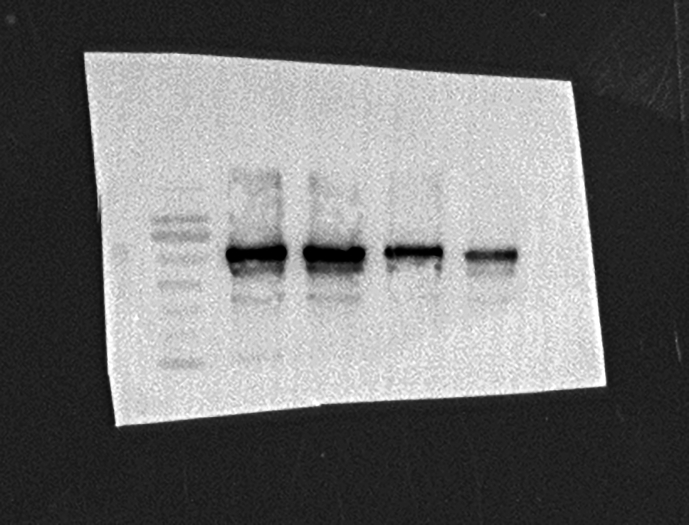


**figs21:**